# Personalized versus standard cognitive behavioral therapy for fear of cancer recurrence, depressive symptoms or cancer-related fatigue in cancer survivors: study protocol of a randomized controlled trial (MATCH-study)

**DOI:** 10.1186/s13063-021-05657-z

**Published:** 2021-10-12

**Authors:** Susan J. Harnas, Hans Knoop, Floor Bennebroek Evertsz, Sanne H. Booij, Joost Dekker, Hanneke W. M. van Laarhoven, Marije van der Lee, Ellen Meijer, Louise Sharpe, Mirjam A. G. Sprangers, Annemieke van Straten, Sonja Zweegman, Annemarie M. J. Braamse

**Affiliations:** 1 Department of Medical Psychology, Cancer Center Amsterdam, Amsterdam Public Health Research Institute, Amsterdam UMC, University of Amsterdam, Amsterdam, the Netherlands; 2 Department of Medical Psychology, Amsterdam Public Health Research Institute, Amsterdam UMC, University of Amsterdam, Amsterdam, the Netherlands; 3 Department of Developmental Psychology, Faculty of Behavioural and Social Sciences, University of Groningen, Groningen, the Netherlands; 4 Center for Integrative Psychiatry, Lentis, Groningen, the Netherlands; 5 Department of Psychiatry, Cancer Center Amsterdam, Amsterdam Public Health Research Institute, Amsterdam UMC, VU University, Amsterdam, the Netherlands; 6 Department of Medical Oncology, Cancer Center Amsterdam, Amsterdam UMC, University of Amsterdam, Amsterdam, the Netherlands; 7 Research Department, Center for Psycho-Oncology, Helen Dowling Institute, Bilthoven, the Netherlands; 8 Department of Medical and Clinical Psychology, Center of Research on Psychological and Somatic disorders (CoRPS), Tilburg University Tilburg School of Social and Behavioral Sciences, Tilburg, the Netherlands; 9 Department of Hematology, Cancer Center Amsterdam, Amsterdam UMC, Vrije Universiteit Amsterdam, Amsterdam, the Netherlands; 10 School of Psychology, Faculty of Science, University of Sydney, Sydney, New South Wales Australia; 11 Department of Clinical, Neuro and Developmental Psychology & Amsterdam Public Health Research Institute, VU University, Amsterdam, the Netherlands

**Keywords:** Psychological symptoms, Fear of cancer recurrence, Depression, Cancer-related fatigue, Personalized treatment, Cognitive behavioral therapy, Ecological momentary assessment (EMA), Cancer survivors

## Abstract

**Background:**

Fear of cancer recurrence, depressive symptoms, and cancer-related fatigue are prevalent symptoms among cancer survivors, adversely affecting patients’ quality of life and daily functioning. Effect sizes of interventions targeting these symptoms are mostly small to medium. Personalizing treatment is assumed to improve efficacy. However, thus far the empirical support for this approach is lacking. The aim of this study is to investigate if systematically personalized cognitive behavioral therapy is more efficacious than standard cognitive behavioral therapy in cancer survivors with moderate to severe fear of cancer recurrence, depressive symptoms, and/or cancer-related fatigue.

**Methods:**

The study is designed as a non-blinded, multicenter randomized controlled trial with two treatment arms (ratio 1:1): (a) systematically personalized cognitive behavioral therapy and (b) standard cognitive behavioral therapy. In the standard treatment arm, patients receive an evidence-based diagnosis-specific treatment protocol for fear of cancer recurrence, depressive symptoms, or cancer-related fatigue. In the second arm, treatment is personalized on four dimensions: (a) the allocation of treatment modules based on ecological momentary assessments, (b) treatment delivery, (c) patients’ needs regarding the symptom for which they want to receive treatment, and (d) treatment duration. In total, 190 cancer survivors who experience one or more of the targeted symptoms and ended their medical treatment with curative intent at least 6 months to a maximum of 5 years ago will be included. Primary outcome is limitations in daily functioning. Secondary outcomes are level of fear of cancer recurrence, depressive symptoms, fatigue severity, quality of life, goal attainment, therapist time, and drop-out rates. Participants are assessed at baseline (T0), and after 6 months (T1) and 12 months (T2).

**Discussion:**

To our knowledge, this is the first randomized controlled trial comparing the efficacy of personalized cognitive behavioral therapy to standard cognitive behavioral therapy in cancer survivors. The study has several innovative characteristics, among which is the personalization of interventions on several dimensions. If proven effective, the results of this study provide a first step in developing an evidence-based framework for personalizing therapies in a systematic and replicable way.

**Trial registration:**

The Dutch Trial Register (NTR) NL7481 (NTR7723). Registered on 24 January 2019.

## Background

Depressive symptoms, fear of cancer recurrence, and cancer-related fatigue are prevalent symptoms among cancer survivors. It is estimated that 22–87% of cancer survivors report moderate to severe fear of cancer recurrence and 8–21% of cancer survivors meet criteria of depression, based on diagnostic interviews or exceeding a cut-off of clinically significant symptoms on self-report measurements [1, 2]. Severe fatigue is also highly prevalent during and after cancer treatment, in at least a quarter of cancer survivors [3, 4]. Presence of fatigue, depression, and/or fear of cancer recurrence is associated with impaired quality of life and daily functioning. In addition, depressive symptoms, fear of cancer recurrence, and fatigue often co-occur and can exacerbate each other [1–3, 5, 6].

Previous studies evaluated interventions for fear of cancer recurrence, depression, and fatigue in cancer survivors or patients with cancer [7–12]. The vast majority of these interventions are based on a cognitive behavioral model which assumes that although cancer and cancer treatment may trigger the psychological symptoms and fatigue, cognitive and behavioral factors subsequently perpetuate the symptoms [7, 8, 11]. Interventions targeting these perpetuating factors have been shown to decrease symptoms and improve functioning. However, effect sizes are mostly small and sometimes of a moderate magnitude, especially for the treatments aimed at fear of cancer recurrence and depressive symptoms [7, 10, 12]. Also, treatment effects are not always sustained at long-term follow-up [8] and attrition rates are relatively high [13].

Tailoring interventions to specific patient characteristics and needs is assumed to improve treatment effectiveness and increase treatment engagement and adherence [14]. Therefore, there has been a repeated call for the personalization of psychological interventions for cancer patients [15–17]. Despite its intuitive appeal, there is, however, insufficient evidence to support this assumption. A few studies have applied personalization in cognitive behavioral therapy (CBT) for cancer survivors or patients with cancer [18–28]. However, to the best of our knowledge, only one of these studies made a head-to-head comparison between the efficacy of personalized CBT to standard CBT [19]. Results from this study, in which a CBT program was tailored by using diagnostic profiles in patients with cancer-related pain, showed greater improvement in patients receiving personalized CBT immediately after treatment, 1 month after treatment and at 6 months follow-up. To determine if personalized CBT has added value over standard CBT, more studies are needed in which a head-to-head comparison is made. Therefore, in this study, we compare the efficacy of personalized CBT to standard CBT in cancer survivors with moderate to severe fear of cancer recurrence, depressive symptoms and/or cancer-related fatigue.

CBT can be personalized in several ways and on various dimensions. In this study, CBT is personalized on multiple dimensions in order to create maximal contrast between personalized and standard CBT: (1) the allocation of treatment modules based on ecological momentary assessments, (2) treatment delivery (face-to-face, internet-based or blended), (3) patients’ needs regarding the symptom for which they want to receive treatment, and (4) treatment duration. Below, these dimensions are described in more detail.

### Allocation of treatment modules

In most intervention studies so far, all patients receive the same treatment protocol, consisting of a fixed set of treatment modules. One of the research directions in the field of personalizing CBT has focused on allocating treatment modules to individual patients [29]. For example, in an intervention for cancer-related fatigue in cancer survivors, the presence of potential maintaining factors is identified with corresponding questionnaires. If a maintaining factor appears to be present, the associated treatment module is added to the treatment plan [30, 31]. Although personalized to some extent, the norm scores used to determine if a treatment module should be allocated are still based on nomothetic (i.e., group-based) research data. Further, the presence of a specific maintaining factor does not automatically imply a causal relationship between the maintaining factor (e.g., a disturbed sleep-wake rhythm) and the symptom of interest (e.g. cancer-related fatigue). Innovative technologies such as ecological momentary assessments (EMA) can establish temporal associations between (maintaining) factors and symptoms of interest [32]. EMA delivers personalized and contextualized information. Therefore, EMA is suited for systematically personalizing interventions and adjusting treatment on an individual level. As the specific maintaining factors in a specific individual can be identified and targeted, the associated treatment modules can be more confidently assigned to the patient.

### Treatment format

The format in which the treatment is delivered can also be personalized. Currently, most research studies include only one treatment form: patients receive either face-to-face, Internet-based, or a mix of these, called blended treatment. In order to provide care adjusted to individual patient’s needs, patients should be able to choose one of these three formats [33].

### Patients’ needs

Allowing patients to choose their most burdensome symptom for which they want to receive psychological treatment seems relevant in personalizing treatment. As previously mentioned, psychological symptoms often co-occur and previous research showed that the presence of clinically relevant levels of a psychological symptom does not automatically imply a need for psychological care for this symptom [34–37]. For example, depressive symptoms can be severe, but not causing hindrance or disability. Personalization could mean that psychological treatment focuses on treating the most burdensome symptom, for which the patient would like to receive psychological care. A previous meta-analysis indicated that following patients’ treatment preferences was associated with lower dropout rates [38].

### Treatment duration

Previous studies showed that there are large individual differences in how quickly patients respond to psychological interventions [39, 40]. Therefore, a standard treatment duration is not always appropriate. Patients could be actively involved in the decision when to end treatment. For example, treatment may be ended when patients feel to have improved sufficiently or to a “good enough level” [39]. This level could be determined by both the patient and the therapist, for example by evaluating to what extent goals have been reached or by evaluating the progress that has been made. Treatment duration and/or focus of treatment could be adjusted accordingly.

### Aims of the current study

The aim of this study is to investigate if personalized CBT is more efficacious than standard CBT in cancer survivors with moderate to severe fear of cancer recurrence, depressive symptoms, and/or cancer-related fatigue. Primary outcome is limitations in daily functioning. Secondary outcomes are symptoms of fear of cancer recurrence, depression, and cancer-related fatigue; quality of life; goal attainment; drop-out rates; and therapist time needed for treatment delivery. We hypothesize that personalized CBT, in this study defined as an intervention tailored to characteristics and needs of the individual patient, will lead to fewer limitations in daily functioning than standard CBT. We also expect that personalized CBT will lead to less symptoms of fear of cancer recurrence, depression, and cancer-related fatigue; better quality of life; higher goal attainment scores; and lower drop-out rates than standard CBT. Finally, we hypothesize that personalized CBT will not increase the time spent by therapists to deliver the intervention.

## Methods/design

The study protocol is reported in accordance with the SPIRIT statement for randomized trials [41].

### Study design

**Fig. 1:**
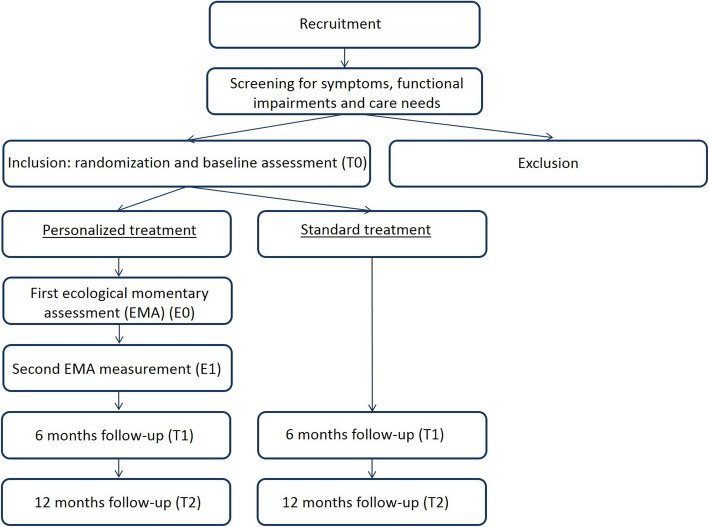
Study design

This randomized controlled trial is designed as a non-blinded, parallel group, two-arm superiority trial with a 1:1 allocation ratio. The two treatment arms are (a) personalized cognitive behavioral therapy and (b) standard cognitive behavioral therapy. Patients in the standard treatment arm receive an evidence-based protocolized CBT for depressive symptoms, fear of cancer recurrence, or cancer-related fatigue. Patients in the personalized treatment arm also receive CBT for depressive symptoms, fear of cancer recurrence, or cancer-related fatigue. However, the treatment in the personalized treatment arm is systematically tailored to the individual. Tailoring takes place on four dimensions: (1) the allocation of treatment modules, (2) treatment delivery (face-to-face, internet-based or blended), (3) patients’ needs regarding the symptom for which they want to receive treatment, and (4) treatment duration. Primary outcome (limitations in daily functioning) and secondary outcomes (symptom level and quality of life) are evaluated at baseline (T0) and 6 months (T1) and 12 months (T2) after intake. At the end of treatment, goal attainment is evaluated and therapist time is assessed. Drop-out rates are measured during the study. The overall study design is illustrated in Fig. 1. The study protocol has been approved by the Medical Ethics Committee of Amsterdam UMC, University of Amsterdam, Amsterdam, the Netherlands (reference no. 2018_277). The study is registered in the Dutch Trial Registry (NTR): NL7481 (NTR7723).

### Recruitment

Patients are referred for participation in this study via their treating physician or via self-referral.

#### Referrals by medical professionals

Patients are recruited by medical professionals (physicians and nurses) at the outpatient cancer clinics of four academic hospitals and four general hospitals in the Netherlands. Medical professionals verbally inform eligible patients about the study during regular medical follow-up consultations. If a patient agrees to be contacted by the researcher for further information about the study, the medical professional sends the contact information to the researcher. The researcher calls the patient to give a detailed explanation about the study and addresses questions. An information package is sent by mail to the patient, including the informed consent form. Patients are asked to sign and return the informed consent form in order to participate in the study, after a reflection period of 7 days.

Patients are also recruited through a psycho-oncological mental health care center (Helen Dowling Institute, Bilthoven) and a tertiary treatment center for chronic fatigue (Nederlands Kenniscentrum voor Chronische Vermoeidheid, Amsterdam). Eligible patients are identified and informed about the study by employees of the two centers. If a patient agrees to be informed about the study by the researcher, the aforementioned procedure for referral and obtaining informed consent is followed.

#### Self-referrals

Patients are also informed about the study by leaflets and notifications on social media of cancer patient associations in the Netherlands. Interested patients can contact the research team by phone or e-mail. Subsequently, the researcher contacts patients by phone to inform them about the study and to address questions. An information package is sent by mail to the patient, including the informed consent form. Patients are asked to sign and return the informed consent form after a reflection period of 7 days in order to participate in the study.

### Participant eligibility

After receiving a signed informed consent form, potential participants are screened for eligibility. The in- and exclusion criteria are shown in Table 1. To verify the medical criteria of self-referrals (cancer diagnosis, time since end of treatment and disease status), patients are asked to send a copy of the necessary medical information of their medical file to the research team. If a patient asks the research team to contact the treating physician, this is done after receiving consent from the patient.

**Table 1:** In- and exclusion criteria

Inclusion criteria	
1)	≥ 18 years old.
2)	Able to speak and read Dutch.
3)	Previously diagnosed with cancer.
4)	At least 6 months and maximum 5 years after end of primary treatment with curative intent. Patients who still receive hormonal therapy after treatment are eligible.
5)	No disease activity at time of inclusion in the study.
6)	Report either clinically relevant levels of fatigue (Checklist Individual Strength (CIS), cutoff ≥ 35 on the fatigue severity subscale), and/or fear of cancer recurrence (Cancer Worry Scale (CWS), cutoff ≥ 10), and/or depressive symptoms (Beck Depression Inventory Primary Care (BDI-PC), cutoff ≥ 4), from which they experience functional impairments (Work and Social Adjustment Scale (W&SAS), cutoff ≥ 10
Exclusion criteria	
1)	Insufficient command of the Dutch language.
2)	Currently receiving psychological or psychiatric treatment.
3)	No informed consent.

To verify the other criteria, the research team administers an online screening questionnaire, screening for severe fatigue (Checklist Individual Strength (CIS) [42, 43]), depressive symptoms (Beck Depression Inventory for Primary Care (BDI-PC) [44, 45]), fear of cancer recurrence (Cancer Worry Scale (CWS) [46]), and impairments in daily functioning (Work and Social Adjustment Scale (W&SAS) [47, 48]). The research team also checks if the patient is currently receiving psychological or psychiatric treatment, as this is an exclusion criterion. The research team verifies the patient’s most burdensome symptom for which he/she would like to receive psychological treatment in the study. In case patients score below the cutoff score on the corresponding symptom questionnaire (e.g., fatigue) but above the cutoff on one or both of the other two symptoms (e.g., fear of cancer recurrence), patients are informed about this and provided with the option of receiving treatment for the latter symptom. If patients do not want this, they are referred to usual care.

### Protocol amendments

Recruitment of participants for this study started in February 2019. We initially aimed to include three patient groups known to have a high symptom burden: patients with esophageal cancer treated with esophagectomy [49], patients with hematological malignancies treated with allogeneic stem cell transplantation [50], and breast cancer survivors [5]. However, recruitment rates were lower than expected during the first year, which led us to broadening our inclusion criteria. As psychological symptoms and fatigue, as well as the factors maintaining these symptoms, are expected to be individual rather than diagnosis-specific, we opened the study for all tumor types, with approval from the funder and the Medical Ethics Committee of Amsterdam UMC, University of Amsterdam, Amsterdam, the Netherlands. As a consequence, we removed the original stratification for cancer type from our randomization procedure. We also broadened our criterion regarding time since end of treatment from 3 to 5 years, following the advice of collaborating medical professionals. All study protocol amendments have been reviewed and approved by the Medical Ethics Committee of Amsterdam UMC, University of Amsterdam, Amsterdam, the Netherlands.

### Assessment

**Fig. 2:**
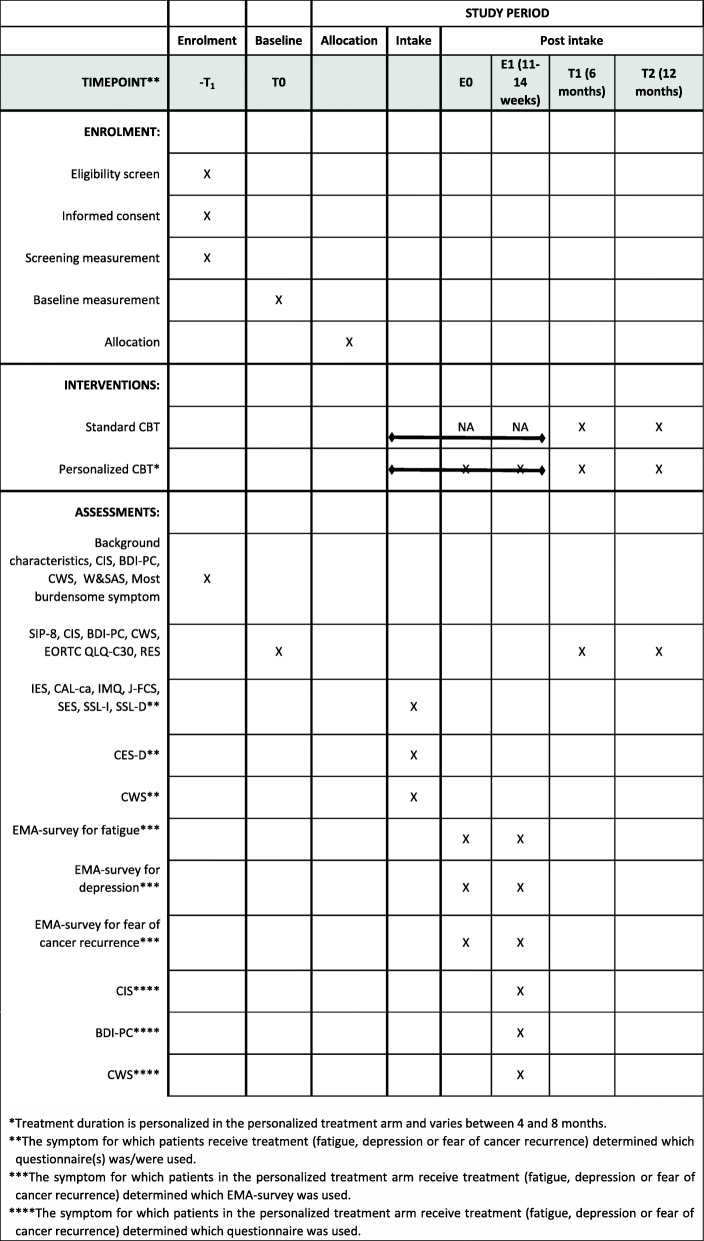
Time points of all measurements

Eligible patients are asked to complete a baseline assessment (T0) before the start of the intervention and follow-up assessments at 6 months (T1) and 12 months (T2) after intake. If the intervention takes >6 weeks shorter or longer than the 6 month T1 assessment, the T1 assessment will be completed as planned, and an additional assessment including the primary outcome and appropriate symptom questionnaire will be completed at the end of the intervention. Outcomes and other study parameters are gathered by the research team using online data collection forms and self-report questionnaires hosted by Castor EDC [51]. In the self-report questionnaires, it is not possible for patients to skip questions. Patients in the personalized treatment arm additionally complete ecological momentary assessments (EMA) at the start of treatment (E0) and during treatment (E1). Because the modules of the three treatment protocols differ in number and duration, the E1 assessment is completed after four treatment modules in the intervention for cancer-related fatigue (± 14 weeks) and fear of cancer recurrence (± 11 weeks), and after five treatment modules in the intervention for depressive symptoms (± 13 weeks). This way, all patients have completed the first mandatory modules and at least one optional module at the E1 assessment (see also section “Personalized CBT protocol”). See Fig. 2 for the assessments at all time points.

#### Participant retention

Participants receive the screening, baseline (T0), and follow-up assessments (T1 and T2) online. Participants are randomized after completing all questionnaires at screening and baseline (T0), preventing missing data at these important moments. Also for optimal retention, the therapists announce the first follow-up assessment to participants at the end of treatment. The research assistant also contacts the participants by email or phone to introduce the last follow-up assessment. Lastly, participants will be contacted by email or phone by the research assistant if they have not completed the questionnaires within 1 week. This applies to all assessments.

#### Handling and storage of data and documents

Personal data will be handled confidentially and in a coded way and comply with the Dutch Personal Data Protection Act (in Dutch: De Wet Bescherming Persoonsgegevens, Wbp). Patient identification will be coded for all study procedures. Only researchers directly involved in the project are allowed to access the codes and participant data. Codes and participant data will be stored in password-protected files. After finishing the study, the key to the code will be safeguarded by the coordinating investigator. Data will be stored by the Department of Medical Psychology of Amsterdam UMC, location AMC for 15 years following completion of the project. Professional secrecy and confidentiality will be maintained at all times. Data, other than the questionnaires, are entered in an electronic case report file (eCRF), which includes an audit trail.

#### Monitoring and auditing

This study will be subject to on-site monitoring in accordance with the quality assurance advice of the Netherlands Federation of University Medical Centres (NFU) regarding research involving human subjects. On-site monitoring will be based on the risk-classification (negligible). At the time of our approval of the Medical Ethics Committee, it was established that due to the low risk of our study, a data monitoring committee was not needed. The research team will submit a summary of the progress of the trial to the Medical Ethics Committee of Amsterdam UMC, University of Amsterdam, Amsterdam, the Netherlands once a year. Information will be provided on the date of inclusion of the first subject, numbers of subjects included and numbers of subjects that have completed the trial, serious adverse events/ serious adverse reactions, other problems, and amendments.

### Outcomes

#### Primary outcome

Limitations in daily functioning are measured with the Sickness Impact Profile-8 (SIP-8) [52]. This questionnaire measures limitations in daily functioning across eight domains: sleep and rest, homemaking, mobility, social interactions, ambulation, leisure activities, alertness behavior, and work limitations. The scores across the eight domains are summed to provide one weighted score of limitations in daily functioning (SIP-8 total score). Higher scores reflect higher levels of functional impairment. The SIP yields sufficient levels of reliability and content validity [53]. As severity and type of psychological symptoms in this study will differ among patients (fear of cancer recurrence, depressive symptom, cancer-related fatigue) and mere presence of psychological symptoms is not an indicator for psychological treatment, limitations in daily functioning are the primary outcome of this study. The SIP-8 total score is measured at baseline (T0), end of treatment (T1), and 1 year after start of treatment (T2). The mean difference on the SIP-8 (total score) from T0 to T1 and T2 is compared between both groups.

#### Secondary outcomes

*Fatigue severity* is measured with the subscale fatigue severity (8 items, 7-point Likert scale) of the Checklist Individual Strength (CIS-fatigue, 20 items) [42, 43]. Scores of this subscale range from 8 to 56. A score of 35 points or higher is an indication for severe fatigue. The CIS-fatigue has previously been used in intervention studies, has been proven reliable and sensitive to change, and has good discriminative validity [31, 54, 55]. Fatigue severity is measured at T0, T1, and T2. The mean difference of fatigue severity (total score on subscale) from T0 to T1 and T2 is compared between both groups.

*Depressive symptoms* are measured with the Beck Depression Inventory for Primary Care (BDI-PC), with a cutoff of ≥ 4 [44, 45]. This 7-item self-report instrument is regularly used to measure depression severity in patients with somatic diseases and has shown to be valid, reliable, and responsive to change [56]. Depressive symptoms are measured at T0, T1, and T2. The mean difference on the BDI-PC (total score) from T0 to T1 is T2 is compared between both groups.

*Fear of cancer recurrence* is measured with the Cancer Worry Scale (CWS). This 6-item questionnaire has been validated as a screening instrument and is able to detect highly fearful patients from non-fearful patients [46]. A cut-off score of ≥ 10 appeared optimal for differentiating highly fearful patients from non-fearful patients. The CWS has good psychometric properties [46]. Fear of cancer recurrence is measured at T0, T1, and T2. The mean difference on the CWS (total score) from T0 to T1 and T2 is compared between both groups.

*Quality of life* is measured with the EORTC QLQ-C30 version 3.0. This is a cancer-specific health-related quality of life questionnaire [57]. The questionnaire incorporates five functional scales, three symptom scales, a global health status / quality of life scale, and a number of single items assessing additional symptoms. For this study, the scales for global quality of life, emotional functioning, role functioning, and fatigue are used. The instrument has been shown to be valid, reliable, and responsive to change [57]. Quality of life is measured at T0, T1, and T2. The mean difference on the EORTC QLQ-C30 version 3.0 (total score) from T0 to T1 and T2 is compared between both groups.

*Goal attainment* is measured with the Goal Attainment Scaling (GAS) procedure. GAS is a method of scoring the extent to which patient’s individual goals for the treatment are achieved. Patients will a priori (at the start of psychological treatment) establish individual criteria for a successful outcome. Each goal is rated on a 5-point scale, with the degree of attainment captured for each goal area. Then, goals are weighted according to their difficulty, and eventually, after end of treatment, goal attainment is evaluated. For details, see Turner-Stokes [58]. This assessment method is included as it concerns a personalized outcome measurement, in which treatment is evaluated based on outcomes specifically relevant and important to the individual patient. Goal attainment is measured at the end of treatment. At this time point, the mean score is compared between both groups.

Therapists register their *time spent on each patient during treatment*. This includes preparation time, registration time in the patient data files (summarizing session), the number of sessions, and time needed to formulate e-consults or feedback in Internet-based or blended therapy. Therapist time is assessed after end of treatment. At this time point, the average time is compared between both groups.

*Drop-out rates* are collected for both treatment arms. In this study, a drop-out is defined as patients who discontinue the intervention before the planned end of the intervention. Patients’ reasons for discontinuing the intervention or declining to fill out follow-up questionnaires will be collected. Drop-out rates at T1 and T2 are compared between both groups.

#### Other study parameters

*Resilience* will be taken into account as a possible moderator. It is assessed with the Resilience Evaluation Scale (RES) [59], which measures five characteristics of resilience: meaning and purposeful life, perseverance, equanimity, self-reliance, and existential aloneness. The RES has 14 items and employs a 7-point Likert type response format. Items scores are summed to yield a total score ranging from 14 to 98, with higher scores indicating greater perceived resilience. Total scores are categorized as very low (14–56), low (65–73), moderate (74–81), moderately high (82–90), and high (91–98) [59].

*Medical information*. The following data is extracted from patients’ medical files: cancer diagnosis, type of medical treatments received, date of diagnosis, date of end of treatment, and disease status (i.e., remission, relapse).

### Randomisation, blinding, and treatment allocation

After completion of the baseline assessment, each patient is randomly assigned by the researcher (SH) or research assistant, who both are not involved in patient treatment, to either the personalized or standard treatment arm. For the randomization procedure, we use a variable block randomization model of an electronic data capture system (Castor EDC), with a 1:1 allocation using variable block sizes of 4, 6, or 8. Stratification by treatment center and referral type is applied. Participants and therapists are blinded for the randomization process, but not for the randomization outcome. Statistical analysis will be done by a statistician blinded for randomization outcome.

At the start of inclusion, 3 treatment centers and 7 therapists participated. These therapists were randomly allocated to either the personalized treatment arm or the standard treatment arm. For allocation, we took treatment center into account, as every participating center needed at least one therapist for the standard treatment arm and one therapist for the personalized treatment arm. Also, we took years of work experience into account to reach an equal distribution of work experience between the two groups as possible (more and less experienced). We divided the 7 therapists (anonymized) in two groups (group A and B) before the randomization, taking the two factors mentioned above into account (treatment center and work experience). The toss of a coin by an independent researcher determined which group was the intervention group and the control group.

#### Procedure for additional treatment sites

During the inclusion period, more treatment centers and therapists were added to the study. To keep an equal distribution of work experience between the two groups and always have one therapist in the personalized treatment arm and one therapist in the standard treatment arm per center, therapists were added to a group based on the distribution as established at the start of inclusion. If a therapist went with maternity leave or stopped participating in the study because of a new job, a new therapist was trained to replace him or her.

### Interventions

#### Standard CBT

In the standard treatment arm, patients receive one evidence-based diagnosis-specific treatment protocol for treating fear of cancer recurrence, depression, or cancer-related fatigue [11, 31, 60–62]. The therapists in the standard treatment arm are not provided with explicit instructions about how to determine which treatment protocol to deliver if a patient is eligible for receiving more than one treatment protocol, based on his/her scores on the screening questionnaires. At the end of the intake, it has to be determined which treatment protocol will be delivered. The psychological treatment is offered in a blended format, which means a combination of face-to-face and internet-based treatment. In Table 2, we describe the procedure in the standard, non-personalized psychological treatment arm.

**Table 2:** Procedure in the standard, non-personalized psychological treatment arm

1)	Patient is referred for participation in this trial via their treating doctor or via self-referral.
2)	Screening, baseline assessment (T0), and randomization.
3)	Intake interview. Following the standard protocols of the treatment for fear or cancer recurrence, fatigue, or depression, additional questionnaires need to be filled out.
4)	Results of additional assessments as part of the standard protocol are discussed.
5)	Start blended therapy for the symptom for which the score on the accompanying questionnaire is above the cutoff.
6)	Patient receives the evidence-based diagnosis-specific treatment protocol.
7)	At the end of treatment (approximately 6 months after intake), follow-up assessment (T1).
8)	12 months after intake, follow-up assessment (T2).

#### Personalized CBT

In the personalized treatment arm, patients receive a personalized version of one of the evidence-based diagnosis-specific treatment protocols. Treatment is systematically personalized on four dimensions:

*Most burdensome symptom*: the goal of this dimension of personalization is to explicitly discuss with the patient from which symptom the patient experiences the most burden, and for which symptom the patient would like to receive treatment. Patients can receive treatment for a symptom, if they score above the predetermined cutoff on the corresponding symptom questionnaire. After intake, it is decided based on the preference of the patient which treatment protocol to start.

*Treatment delivery:* patients choose for either face-to-face, Internet-based, or blended treatment. After the intake interview, the psychologist asks the patient what he or she prefers.

*Treatment modules:* specific factors that maintain the treated symptom in a specific individual are identified with EMA. The identified maintaining factor(s) correspond(s) to different treatment modules of the protocol. Thus, based on the EMA analysis, the most relevant treatment modules are selected for the patient. This procedure is further explained in the section “Ecological momentary assessments (EMA).”

*Treatment duration:* treatment response is evaluated based on measurement of symptom level and progression on individual goals (GAS). If the treatment has effect on the maintaining factors, the symptom level is expected to be below the predefined cutoff and the patient will have reached his/her personal treatment goals. The therapist will discuss with the patient that in this case, treatment conclusion is indicated. However, if the patient and the therapist decide that treatment should continue, the results of the second EMA determines which modules to repeat or add to the treatment plan. At the latest, treatment has to end at 8 months after intake. In Table 3, we describe the procedure in the personalized psychological treatment arm.

**Table 3:** Procedure in the personalized psychological treatment arm

1)	Patient is referred for participation in this trial via their treating doctor or via self-referral.
2)	Screening, baseline assessment (T0), and randomization.
3)	Intake interview (verification patients’ most burdensome symptom and patients’ preference for face-to-face, blended, or Internet therapy). Following the standard protocols of the treatment for fear or cancer recurrence, fatigue, or depression, additional questionnaires need to be filled out.
4)	Start of EMA assessment during 14 consecutive days. Auto-VAR analyses to identify the most important maintaining factor on which to intervene first, besides the mandatory modules.
5)	Results of EMA assessment and additional questionnaires are discussed.
6)	Start treatment aimed at maintaining factors for the most burdensome symptom.
7)	Evaluation of treatment response through measurement of symptom level and progression on individual treatment goals. The therapist discusses with the patient whether treatment continuation or treatment conclusion is indicated. If treatment continues, the second EMA assessment determines which treatment modules to add or repeat.
8)	6 months after intake, follow-up assessment (T1); if treatment continues additional assessment at end of treatment.
9)	12 months after intake, follow-up assessment (T2).

#### Adherence and treatment integrity

Therapists are trained in the treatment protocols by experienced clinical psychologists. Adherence to intervention protocols and treatment integrity will be evaluated with registration forms, on which therapists register which treatment components were discussed during each treatment session, as well as the time they spent on each session. The supervision sessions in group format will also improve treatment integrity, as cases are discussed in the presence of the same experienced clinical psychologists who provided the training. Lastly, drop-outs from the intervention are registered.

### Development process

#### Personalized CBT protocol

Patients in the personalized treatment arm receive personalized treatment for fear of cancer recurrence, depression of cancer-related fatigue. The evidence-based protocols for treating fear of cancer recurrence, depression or cancer-related fatigue were used as a starting point for our personalized version of the treatment [11, 31, 60–62]. The evidence-based protocols were divided into different modules, based on the maintaining factors these modules aim at. This resulted in three to four general modules, essential for every patient, and several optional modules, which will be only provided to patients with these maintaining symptoms. The outcomes of the EMA analyses are used to identify the relevant optional modules for each patient. Table 4 presents the modules per protocol. Experienced cognitive behavioral therapists were asked to provide feedback on the concept protocol, which was processed and led to the definitive treatment protocol. This includes a treatment manual with a detailed description, a format for a personalized treatment plan, and session checklists for each therapy module.

**Table 4:** Treatment modules and maintaining factors per protocol

Protocol	Module	Maintaining factor(s)	Mandatory/Optional
*Fear of cancer recurrence (FCR)*			
	Psycho-education	-	Mandatory
	Values, goals, and actions	-	Mandatory
	Attention training	Self-focused attention	Mandatory
	Detached mindfulness and worry postponement	(1) Worry and rumination (2) Attempts to avoid, suppress, or minimize FCR thoughts (3) Intrusive thoughts or images about cancer recurrence	Optional
	Metacognitions	Unhelpful beliefs about the importance, impact and control of worry	Optional
	Threat-monitoring and avoidance	(1) Frequent self-examination (2) Avoidance behaviors	Optional
	Evaluation and relapse prevention plan	-	Mandatory
*Depression*			
	Psycho-education	-	Mandatory
	Goal setting	-	Mandatory
	Activity monitoring	Behavioral inactivity	Optional
	Activity scheduling		
	Behavioral activation		
	Identifying dysfunctional thoughts	Dysfunctional cognitions	Optional
	Changing dysfunctional thoughts (part 1)		
	Changing dysfunctional thoughts (part 2)		
	Relapse prevention	-	Mandatory
*Fatigue*			
	Psycho-education and goal setting	-	Mandatory
	Sleep-wake cycle	Deregulated sleep-wake cycle	Mandatory
	Activity pattern	Deregulation of activity	Mandatory
	Helping cognitions	Dysfunctional cognitions regarding fatigue	Optional
	Fear of cancer recurrence	Fear of cancer recurrence	Optional
	Social interactions	Low social support	Optional
	Coping	Poor coping with cancer and cancer treatment	Optional
	Realizing treatment goals	-	Mandatory

In addition, the secure web-based environment in which patients receive blended or online treatment was developed (Minddistrict; www.minddistrict.com). The format of existing online therapies was adapted for this specific study.

#### Therapists

A master’s degree in Psychology and clinical experience as a therapist are two minimum requirements for participating as a therapist in the study. All participating therapists are trained in working with the treatment protocols (total of 5 training days) and with the online environment. In addition, therapists allocated to the intervention group are separately trained in working with the four dimensions of personalization in the treatment protocol.

During the study, all therapists (in both groups) receive group supervision by experienced clinical psychologists by phone or video-calls every 2 weeks. To avoid contamination, therapists from the intervention group have supervision sessions separate from the therapists from the control group.

### Ecological momentary assessments (EMA)

#### EMA protocol

For the EMA assessments, an existing online electronic diary system is used, designed to monitor patients in their natural environment (http://roqua.nl). Assessments are prompted five times a day for fourteen consecutive days (E0 and E1), after fixed intervals (every 3 h). The exact time points are adapted to patients’ sleep-wake schedule. Patients receive a text message on their smartphone with a link to a questionnaire. They are asked to fill out the questionnaire immediately after the alert. If the patient does not complete the questionnaire within 30 min after the alert, they receive one reminder text message. One hour after the alert, the questionnaire can no longer be accessed. It takes approximately 2 min to complete the questionnaire and it is not possible to skip questions.

#### EMA questionnaires

To determine which questions to include in the electronic diary, the following steps were followed:

1. Every treatment protocol was divided into multiple modules, based on the maintaining factors these modules aim at. Table 4 presents the individual modules and targeted maintaining factors.
2. EMA items were formulated, based on previous EMA surveys where possible, or on validated, traditional surveys that measured the maintaining factor, in which case the wording of the question was adjusted to reflect the nature of EMA. In case both options were not possible, we formulated the EMA-question ourselves.
3. Three questionnaires were established, one for each treatment protocol, with one or more questions linked to each of the modules and corresponding maintaining factors.
4. The initial set of EMA questions was piloted by a small group of researchers and patients (*n*=4) and adjusted according to the feedback.

#### EMA analyses

The EMA data of individual patients are analyzed directly after obtaining the complete measurements, before (E0) and during treatment (E1). The time series of each individual participant are examined with vector autoregressive (VAR) modeling [63, 64]. Vector autoregressive modeling is especially suitable for investigating the temporal dynamics between two or more time series. In addition, by separating the dynamic part of the model (i.e., the relationships between the time-lagged values of the variables) from the simultaneous part (i.e., the relationships between the contemporaneous variables), the model enables inferences about the temporal order of the effects, also known as Granger causality [32]. Hence, temporal associations between (maintaining) factors and symptoms of interest (fatigue, depression, fear of cancer recurrence) can be established. For the application of this method in clinical practice, the package AutoVAR is used in statistical program R, which encompasses an automated analysis and interpretation of time series data [65]. Before the AutoVAR analyses, the data are checked for missing measurements and, if needed, imputed using the Amelia II package in R [66]. If a variable is skewed, a log- or power transformation is performed before imputation, depending on the direction of the skewness. Next, the mean square successive difference (MSSD) of the variables is checked. If the MSSD is 50 or less, the variable is not included in the AutoVAR analyses. This threshold is used to ensure sufficient variability within each variable and, as such, increases the probability of finding a valid VAR model [67]. A model is considered valid if it passes all the necessary assumptions of a VAR analysis (i.e., stationarity, white noise, homoscedasticity, normality). For more details about the AutoVAR procedure, please see [65]. Then, the AutoVAR analyses are conducted. We apply a maximum lag length of 2 and initially only analyze associations between the targeted symptom and the different maintaining symptoms separate from each other. After the first EMA period, we identify the most relevant maintaining factor, associated with one of the optional treatment modules. The factor that “granger cause” the targeted symptom in the most valid VAR models (highest percentage) is considered to be the most relevant maintaining factor. The associated optional treatment module is added to the treatment plan. If treatment continues after the second EMA period, the analyses of the second EMA determine which treatment modules to add or repeat. Therapists are provided with a report of the individual network of symptoms and maintaining factors as established by the AutoVAR analyses by the primary researcher (SH). This report also includes a treatment plan. For more details about the application of AutoVAR in the MATCH-study please see [68].

#### Back-up questionnaires

It is possible that variables fluctuate too little in some patients, and no valid EMA models can be formed. In this case, the baseline assessment and additional questionnaire(s) are used to identify individual maintaining factors. Patients in both treatment arms fill out the additional questionnaires after the intake. If a patient receives treatment for fear of cancer recurrence, we ask the patient to fill out the 42-item Fear of Cancer Recurrence Inventory (FCRI). Symptoms or issues are rated on a Likert scale from 0 (not at all/never) to 4 (a great deal/all the time). Total scores range from 0 to 168 and a higher score indicates worse fear of cancer recurrence. This questionnaire has sufficient to good reliability and test-retest reliability [69]. The FCRI was also used as primary outcome in the ConquerFear trial [60].

If a patient receives treatment for depressive symptoms, we ask the patient to fill out the 20-item Center for Epidemiological Studies Depression Scale (CES-D). This questionnaire is commonly used in cancer patients and has shown good internal consistency, with alpha coefficients > 0.85 as well as adequate test-retest reliability [70].

If a patient receives treatment for cancer-related fatigue, we ask the patient to fill out additional questionnaires as mentioned in the protocol article of the fatigue intervention [30]. This means the Impact of Event Scale [71], the Modified causal attribution list [72, 73], the Illness management questionnaire [73–75], the Fatigue catastrophizing scale [76], the Self-efficacy scale [73, 77], and the Van Sonderen Social Support Inventory, subscales Interactions (SSLI) and Discrepancies (SSLD) [78]. The psychometric properties of these questionnaires were found to be sufficient.

### Statistical analyses

#### Primary and secondary study parameter(s)

Descriptive statistics will be used to describe baseline characteristics of the experimental and control group and a chi-square test or independent *t*-test will be used to compare baseline characteristics of dropouts and completers in the total sample. Intention to treat analyses will be conducted, i.e., all randomized patients are included and are analyzed in their randomly assigned treatment group. For the intention to treat analyses, linear mixed-model analyses will be performed to evaluate changes in patient functioning from baseline to 6 and 12 months of follow-up (T1 and T2) and differences therein between the experimental group and the control group. Condition, time, and a group × time interaction will be added to the model (as fixed effects) to test for differences between the groups in treatment effects over time. In addition, a random intercept will be used to allow individuals to differ in the level of their outcome variables. Treatment center and manner of referral (self-referral versus referral by treating doctor) will be used as covariates. Based on pooled pre-test standard deviations, effect sizes will be calculated for the estimated differences between T0, T1, and T2 between groups. A significance level of *p*<0.05 is used in all analyses. Since linear mixed model analysis can handle missing observations due to dropout (assuming data are missing at random), imputation of missing values will not be needed. However, patterns of missing data will be explored and predictors of missing data analyzed, especially if these vary by treatment arm. If the primary analysis shows significant effects of personalized CBT, additional sensitivity analyses will be conducted using different assumptions about the value of missing values. Statistical analyses will be performed using SPSS 24.0. The statistical procedure as described above will also be followed for secondary study parameters (fatigue, depressive symptoms, fear of cancer recurrence and quality of life) without the sensitivity analyses. The independent *t*-test will be used to analyze differences between both groups with respect to goal attainment and therapist time.

### Sample size calculation

As linear mixed models will be used for conducting the primary analyses, it is recommended to base the sample size calculation on a multivariate model [79]. Power analyses for MANOVA were conducted with G*power 3.1.9.2. We want to be able to demonstrate an effect size of 0.25 (partial eta squared) on the primary outcome, while setting the alpha = 0.05 (two tailed) and power = 0.80. With two groups and three measurements each, a sample size of 152 is needed. Taking into account a dropout rate of approximately 20%, a sample size of 190 (95 patients per condition) will be included at baseline.

## Discussion

To our knowledge, this is the first study comparing the efficacy of personalized CBT to standard CBT in cancer survivors with moderate to severe fear of cancer recurrence, depressive symptoms, and/or cancer-related fatigue. The study has several innovative characteristics.

The first is the head-to-head comparison between personalized CBT and standard CBT. There has been a repeated call for personalized psychological treatments for cancer patients [15–17]. Multiple studies have applied personalization in cognitive behavioral therapy (CBT) for cancer survivors or patients with cancer [18–28]. However, to the best of our knowledge, only one study compared personalized CBT to standard CBT [19]. In the other studies, personalized CBT was compared to a wait list group or another intervention such as a psycho-education. To determine if and the extent to which personalized CBT has added value over standard CBT in cancer survivors, more studies with a head-to-head comparison are needed. Our study enables a direct comparison.

A second innovative characteristic of this study is the personalization of psychological treatment on various dimensions. Other studies, in which the efficacy of personalized CBT was compared to standard CBT in non-cancer patients, mostly found no significant difference between the two treatment forms. Researchers suggested that insufficient contrast between the two interventions could have partly accounted for the lack of difference [80–82]. Therefore, we aimed to expand the contrast in this study by delivering a package of personalization measures. Should these measures eventually not lead to a difference between treatment arms, the expectations of the added value of personalized psychological treatments should perhaps be reconsidered. However, if personalized CBT appears to be more efficacious than standard CBT, it will remain unclear which personalization measure or combination of personalization measures explains the difference. Further research could then explore the extent to which each personalization measure adds value to personalized CBT. For example, the MOST design could be used to further optimize the intervention [83]. For clarity, we decided to personalize CBT on multiple dimensions to maximize the contrast between personalized CBT and standard CBT.

Another innovative characteristic of this study is the application of EMA in clinical practice, specifically for systematically personalizing CBT. Nowadays, electronic sampling devices provide the opportunity for rapid analysis and availability of real-life experimental data. Recent studies suggested not to use EMA merely as an instrument to monitor, but as a tool to provide feedback and treatment recommendations [32, 63, 84]. In this study, we further concretize how EMA can be used for personalizing psychological treatment in clinical practice, by presenting care providers with prospective information on patients’ psychological symptom patterns, behavior, and contextual influences in daily life, with minimal delay. Individual symptom patterns over time will be identified and thereby the factors that maintain symptoms in specific individuals. The use in clinical practice is promising and a logical next step, but is also new and innovation comes with vulnerabilities. In some patients, symptoms may fluctuate too little, as a result of which no valid EMA models can be formed. For this study, we solved this potential problem by having questionnaires as a back-up. However, as mentioned before, the presence of a maintaining factor identified with a questionnaire does not automatically imply that the factor influences symptom level. Therefore, EMA seems the most desirable approach to determine the maintaining factors, which subsequently inform the direction for treatment.

Previous studies showed that there are large individual differences in how quickly patients respond to interventions [39, 40]. In this study, we aimed to adjust treatment duration on the individual level by adding an “in-between” assessment and evaluating the extent to which treatment goals already have been reached after completing several treatment modules. If treatment continues, the second EMA determines which treatment modules to add or repeat. A previous study in which personalized CBT was compared to standard CBT in a non-cancer population found a beneficial effect of personalized CBT and suggested that this beneficial effect was found because the therapeutic sessions were longer [85]. The current study will provide additional information on the needed treatment duration in personalized CBT compared to standard CBT, and on the extent to which treatment duration differs within the personalized CBT group. Although we will not conduct cost effectiveness analyses, we will have an indication of the costs by therapist time and number of treatment sessions.

In conclusion, this study meets the repeated call for personalized psychological treatment, and the movement towards patient-centered care. This study is an important step, as it will provide much needed evidence regarding if personalized CBT is more efficacious than standard CBT. One could argue that therapists already aim to deliver personalized treatments in clinical practice. However, the challenge is to develop an evidence-based framework for personalizing CBT in a systematic and replicable way [86]. The explicitly operationalized personalization dimensions in this study have the potential to provide the first step in developing this kind of framework.

## Trial status

Protocol version 4.0, 22 January 2021. Recruitment is ongoing.

Recruitment of participants for this study started in February 2019 and is scheduled to finish in June 2022.

## Data Availability

The dataset belonging to this study is not available yet.
